# Quality of TB diagnostic services at primary healthcare clinics in eThekwini district, South Africa

**DOI:** 10.1371/journal.pone.0307149

**Published:** 2024-07-18

**Authors:** Thobeka Dlangalala, Alfred Musekiwa, Tivani Mashamba-Thompson

**Affiliations:** 1 School of Health Systems and Public Health, Faculty of Health Sciences, University of Pretoria, Pretoria, Gauteng, South Africa; 2 Faculty of Health Sciences, University of Pretoria, Pretoria, Gauteng, South Africa; University of Kwazulu-Natal, SOUTH AFRICA

## Abstract

Overcoming the TB epidemic requires moving past expanding the coverage of healthcare services and looking to improve the quality of TB services. During COVID-19, the suboptimal state of TB services has further deteriorated, and little is known about how these services have fared after the pandemic. The study aims to assess the quality TB diagnostic services in primary health care (PHC) clinics in the eThekwini district, South Africa. Twenty-one clinics with the lowest and highest headcounts from each region of eThekwini were purposively selected. An audit tool adapted from the United States Agency for International Development (USAID) and the national TB guidelines was used to collect data on six different audit components. To assess quality, a 3-point scale was used where clinics could get a rating of either excellent, moderate, or poor performance. Descriptive statistics were employed to summarize and analyze clinic scores in Stata v15.1. Additionally, associations between clinic scores and clinic characteristics were investigated using Pearson’s pairwise correlation coefficient and a linear regression model, where p < 0.05 was the measure of statistical significance. The audit found that the quality of diagnostic services in eThekwini was moderate. The gaps that required addressing were the lack of TB training among staff, adherence to infection prevention and control practices, and contact screening. Without feasible solutions, these will hinder current TB management strategies and slow progress toward ending the TB epidemic.

## Introduction

Low- and middle-income countries (LMICs) of sub-Saharan Africa and Asia are the most burdened by Tuberculosis (TB). In 2021, South Africa was one of the countries that made up the largest global TB burden [1]. At the heart of the TB epidemic in South Africa is the high prevalence of HIV within the population and the people who contract TB but are never diagnosed [2]. In 2019, 32 000 people died from TB, of which 62% were HIV positive [3], and in 2021, 304 000 people contracted the illness while the number of deaths increased to 56 000 [4]. The lives of so many people should not continue to be lost to a curable disease in which policies and interventions are in place to assist with its control [5]. Therefore, it is time to investigate the quality of care at facilities providing TB services in LMICs.

Evidence shows that half of TB deaths now occur as a result of poor quality rather than limited access to healthcare [6]. There is agreement among stakeholders that improving TB outcomes must go beyond increasing coverage of healthcare services and shift towards improving the quality of TB care [7, 8]. The Lancet Global Health Commission has published a report that states that providing healthcare services without the base level of quality is unethical, wasteful, and ineffective [9]. The quality of TB care has been assessed in several countries, and the results revealed suboptimal TB care in both the public and the private sectors [10–14]. Similar studies from South Africa that used standardized patients to measure quality showed better TB management than in other countries. However, there was still sub-optimal management of HIV during TB screenings [10, 11]. This is concerning for a country that has integrated TB and HIV services as a means to manage the high HIV, TB, and HIV-TB coinfection prevalence [15].

COVID-19 has further complicated the provision of essential TB care in high-burden countries [16]. During the initial stages of COVID-19, access to TB services was limited due to the introduction of lockdowns and the diversion of resources to help manage the spread of SARS-CoV-2 [17]. This reduced the case detection in TB-endemic countries like South Africa, wherein TB detection dropped by 26% in 2020 compared to 2019 [16]. Subsequently, the reduction in the number of people diagnosed with TB has also increased TB mortality [16]. The ambitious targets the United Nations (UN) and the World Health Organization (WHO) set to eradicate TB are now out of reach [18, 19]. Rapid restoration of TB services and the scale-up of case detection will play a vital role in getting TB goals back on track. However, this would be futile without ensuring quality services at facilities offering care.

Although South Africa has implemented continuous quality improvement to consistently assess the performance of TB and HIV services [20, 21], to our knowledge, no assessments of TB services have been published since the start of the COVID-19 pandemic. For this reason, the study aimed to assess the quality TB diagnostic services at primary healthcare clinics (PHCs) in the eThekwini district, South Africa. A study of this nature would reveal the barriers that will guide targeted implementation strategies aimed at quality improvement.

## Methods

### Study design

A facility audit focused on the quality of TB diagnostic services was conducted at PHCs. An audit allowed us to gain insight into the availability and functionality of the diagnostic resources at the clinics [22]. This study forms part of a larger study that seeks to improve TB diagnostic services at PHCs during pandemics and similar situations [23].

### Study setting

The study was undertaken in eThekwini district, the largest metropolis in Kwa-Zulu Natal province, South Africa. The area is divided into four municipal functional planning regions: the North, South, Central, and Outer West [24]. The population of eThekwini is estimated at 4 million, of which the majority reside in the southern region [24]. A key health challenge within the region is the low life expectancy due to high disease prevalence, including TB [25]. The TB prevalence survey conducted in 2018 reported a prevalence of 737 cases per 100 000 people within the region [26]. Furthermore, TB detection was disrupted by the COVID-19 pandemic pointing to a need for strengthened detection and diagnostic services [27]. The frontline of TB diagnostic services in South Africa are PHCs which serve as the first point of contact for receiving a test in the public sector, which serves 80% of the country’s population [28]. eThekwini currently has 123 PHCs administered through the local authority or provincial government.

### Study population and sampling strategy

The research was conducted at clinics providing TB diagnostic and treatment services in the eThekwini district. eThekwini had a population of approximately 4 million people in 2021, distributed across different regions: 33% in the northern region, 31% in the central region, 25% in the southern region, and 11% in the western region. A sample clustering technique was used to divide clinics into clusters representing each of the four municipal planning regions in eThekwini. Within each cluster purposive sampling was employed to choose clinics with a high patient headcount. In total, 21 clinics were included six of these were from the southern and central regions, five from the northern region, and four from the western region.

### Data collection and scoring guide

An audit assessment tool developed by MEASURE evaluation was used to collect data on TB diagnostic services in eThekwini [29]. The tool was designed to assess the quality of TB services at the facility level to inform action plans that strengthen these services where required [29]. The tool was adapted to focus on the diagnostic aspect of TB services and also represented the priorities set by South Africa’s National Department of Health (NDoH) (S1 Table) [30]. Adaptation of the tool was done through a consultative process with a TB program manager to ensure it captured the national standards of TB diagnostic care. The tool assessed six key components: TB diagnosis and management, HIV-TB integration, TB documents and guidelines, specimen management, infection prevention and control (IPC), and TB training.

The responses on the audit tool were either “yes” or “no,” where “yes” was coded as “1” and “no” as 0. The primary investigator only recorded “yes” responses when clinic personnel could show evidence of the audit item. For items lacking evidence, staff were asked to explain, resulting in a subsequent ‘No’ response. The resulting ‘yes’ responses were totaled and converted to percentages for every component. The overall facility scores were determined by averaging the percentages from each audit component. The performance was determined using three cut-off points: scores of between 85%-100% signified excellent performance, those between 50%-84% were moderate, and scores lower than 50% represented poor performance.

#### Data collection procedure

The facility audits took place over three months, from 12 January to 25 March 2023. The audit team comprised the primary investigator (PI), clinic managers, and a TB nurse from each audited facility. The PI explained the components of the audit tool to the clinic’s respective members. Then, each clinic representative answered the questions on the checklist and showed the PI various items as dictated by the audit checklist. To ensure the audit’s efficiency, staff members were notified of the purposes of the audit before the clinic visit.

#### Improving data quality

The data collection tool was adapted in collaboration with a TB program manager to ensure that the tool encompassed the national priorities for TB diagnostic services. To ensure the correct functioning of the tool, it was pre-tested at a facility that did not form part of the study and adjusted as required. A single person handled data collection to maintain consistency in recording and capturing data. After collection, the data underwent capture and cleaning using Microsoft Excel before analysis.

### Data analysis

Data were collected manually at respective clinics before being entered into an Excel spreadsheet, where it was cleaned and validated. Once clean, data were exported into Stata version 15.1 for analysis [31]. The t-test was used to estimate the frequencies and the 95% confidence intervals for overall audit scores and respective audit components. To compare the statistical differences in the performance of the audited clinics by region, a one-way Analysis of Variance (ANOVA) was conducted and adjusted for multiple comparisons using Tukey’s post-estimation test, where p < 0.05 was significant. The association between the audit scores and clinic characteristics was determined using Pearson’s pairwise correlation coefficient, p < 0.05, as a measure of significance. Variables were then subjected to a linear regression, which used R^2^ to measure the goodness-of-fit and p < 0.05 for statistical significance.

### Ethical considerations

This study received ethical approval from the University of Pretoria’s Health Sciences Research Ethics Committee Reference number 652/2021. Further approvals were obtained from the KwaZulu Natal Department of Health (Ref: KZ_202112_012) and the eThekwini Municipality’s Health Unit. Anonymous patient data were extracted and aggregated for this study; thus, informed consent from patients was waived by the University of Pretoria’s Health Sciences Research Ethics Committee. Written consent was provided by the clinic staff who participated in the audit.

## Results

### Characteristics of audited clinics

A total of 21 PHCs were audited from the eThekwini district, South Africa, over three months between January and March 2023. Five clinics were audited from the northern region, six from the southern and central regions, and four from the western region. Eight out of the 21 audited clinics were administered by the provincial government and the remaining 13 were issued by the local municipal government. Eighteen facilities provided TB-related services from Monday to Friday, one from Monday to Saturday, and only two reported providing these services from Monday to Sunday. Fourteen clinics indicated that TB screening was conducted in all consultation rooms within the facility, one clinic had TB services at three points, two clinics provided these services in two areas, and five clinics provided TB services at a single service point. The average quarterly headcount of the clinics ranged between 9 755±2 578 and 21 405± 9 075 patients among the four regions, while the number of people screened for TB varied between 9 213 ± 2 454 and 18 385 ± 7 924. A detailed description of the audited clinics is found in Table 1.

**Table 1:** Characteristics of the audited PHCs within eThekwini district, South Africa, ± represents the standard deviations.

Region	No. of municipal clinics	No. of provincial clinics	Days per week, TB services are available	No. of TB-related service points*	Patient headcount (Quarterly)	Patients screened for TB symptoms (Quarterly)
**North**	5	0	Monday-Friday	One to all points	9 755 ± 2578	9 213 ± 2 454
**South**	1	5	Monday-Friday	One to all points	21 405 ± 9 075	18 385 ± 7 924
			Monday-Saturday			
			Monday-Sunday			
**Central**	3	3	Monday-Friday	Two to all points	19 208 ± 10 859	16 564 ± 8 540
**West**	4	0	Monday- Friday	One to all points	13 350 ± 5 087	12 590 ± 5 731

*These indicate the range of TB related services points found at clinics within the respective regions of eThekwini district.

### Audit scores for facilities in eThekwini district, South Africa

**Fig 1:**
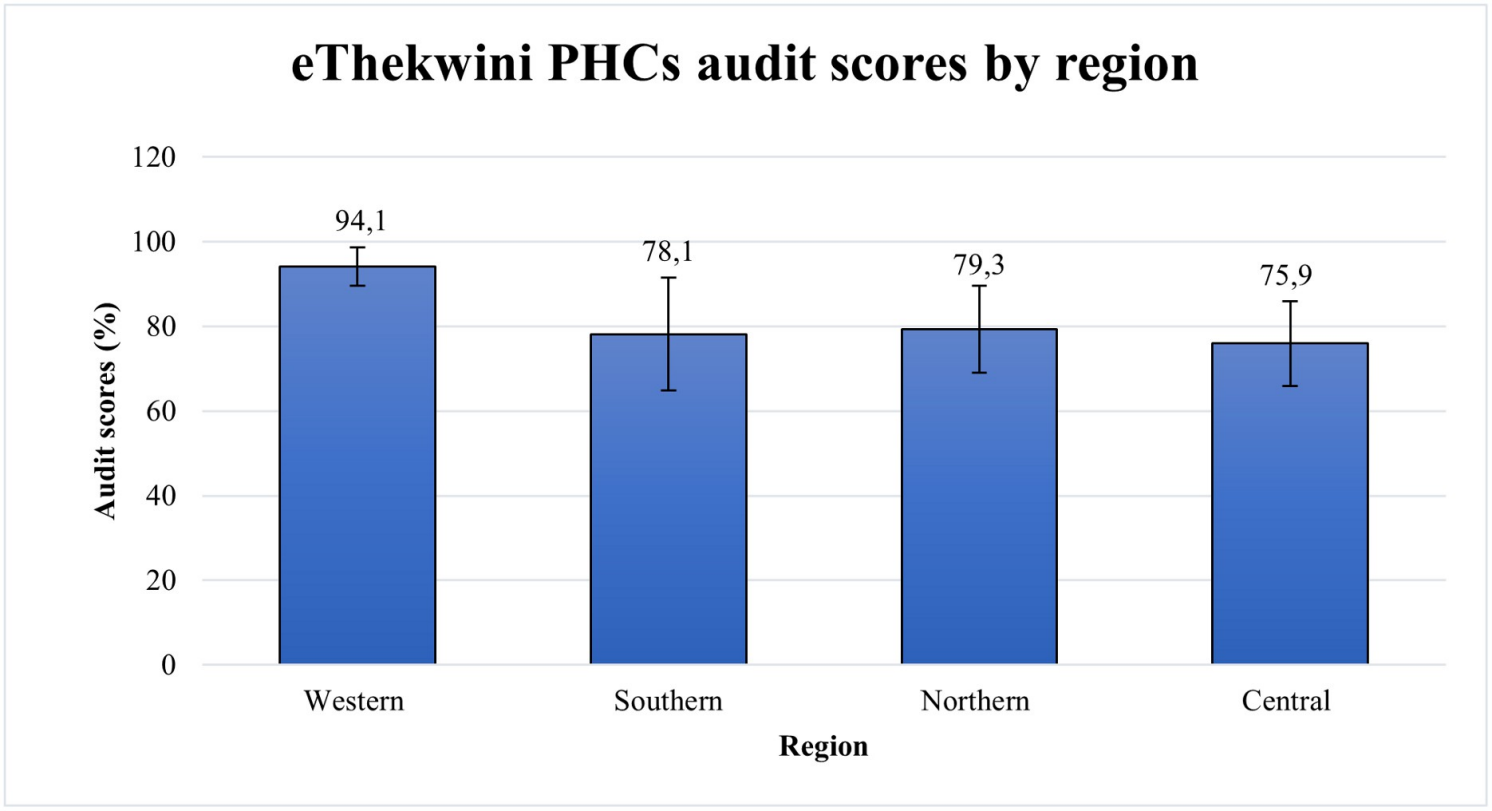
Audit scores for TB diagnostic service at PHCs in eThekwini by region, where error bars represent 95% confidence intervals.

Based on the criterion applied to this research, the results from the audited clinics show individual ratings that range between moderate 64.1% (95% CI: 16.0%-112.3%) and excellent 97.6% (95% CI:91.6%–103.6%) Table 2. Nine out of the 21 clinics scored excellently, and the remaining 12 performed moderately (Table 2). Clinics in the west were the only ones to achieve an excellent rating with a combined score of 94.1%, and conversely, facilities in the central region had the lowest performance at 75.8%; moreover, the difference in scores between these two regions was statistically different (p < 0.05). Clinics of the southern and northern regions shared similar scores of 78.1% and 79.3%, respectively (Fig 1).

**Table 2:** Individual audit scores and ratings for PHCs in the eThekwini district.

		Infection Prevention & Control	Specimen management	Documents & guidelines	TB training	Integration of TB/HIV services	TB diagnosis & management	Overall percentage per clinic (95% CI)	Clinic rating
**Individual clinics represented by region**	**South 1**	86	100	100	100	100	50	89.3 (68.2–110.3)	Excellent
	**South 2**	71	75	57	100	100	40	73.8 (48.9–98.6)	Moderate
	**South 3**	71	100	14	0	100	100	64.1 (16.0–112.3)	Moderate
	**South 4**	86	75	57	0	100	83	66.8 (29.4–104.2)	Moderate
	**South 5**	93	100	86	100	100	100	96.5 (89.4–104.9)	Moderate
	**South 6**	86	100	100	0	100	83	78.1 (37.1–119.1)	Moderate
	**Central 1**	57	75	100	100	100	83	85.8 (67.3–104.3)	Excellent
	**Central 2**	71	100	86	100	100	67	87.3 (71.3–103.3)	Excellent
	**Central 3**	61	75	86	0	100	60	63.6 (27.2–100.0)	Moderate
	**Central 4**	64	100	100	0	100	100	77.3 (34.7–119.8)	Moderate
	**Central 5**	86	100	100	0	20	100	67.6 (19.9–115.3)	Moderate
	**Central 6**	71	100	86	0	100	83	73.3 (33.9–112.7)	Moderate
	**West 1**	64	100	100	100	100	83	91.1 (75.4–106.8)	Excellent
	**West 2**	71	100	100	100	100	100	95.1 (82.7–107.5)	Excellent
	**West 3**	86	100	100	100	100	100	97.6 (91.6–103.6)	Excellent
	**West 4**	86	100	86	100	100	83	92.5 (83.8–101.1)	Excellent
	**North 1**	79	100	71	100	100	50	83.3 (61.7–104.9)	Moderate
	**North 2**	79	100	85	100	100	50	85.6 (65.0–106.3)	Excellent
	**North 3**	71	100	75	100	100	67	78.3 (59.6–97.0)	Moderate
	**North 4**	71	100	86	100	20	75	75.3 (44.1–106.5)	Moderate
	**North 5**	64	100	86	0	100	50	66.6 (26.4–106.8)	Moderate
	**% per component (95% CI)**	74.9 (70.3–79.5)	95.2 (90.6–99.8)	83.8 (74.3–93.3)	61.9 (37.5–82.1)	92.3 (81.4–103.3)	76.5 (67.3–85.7)		

### Performance by individual audit component

**Fig 2:**
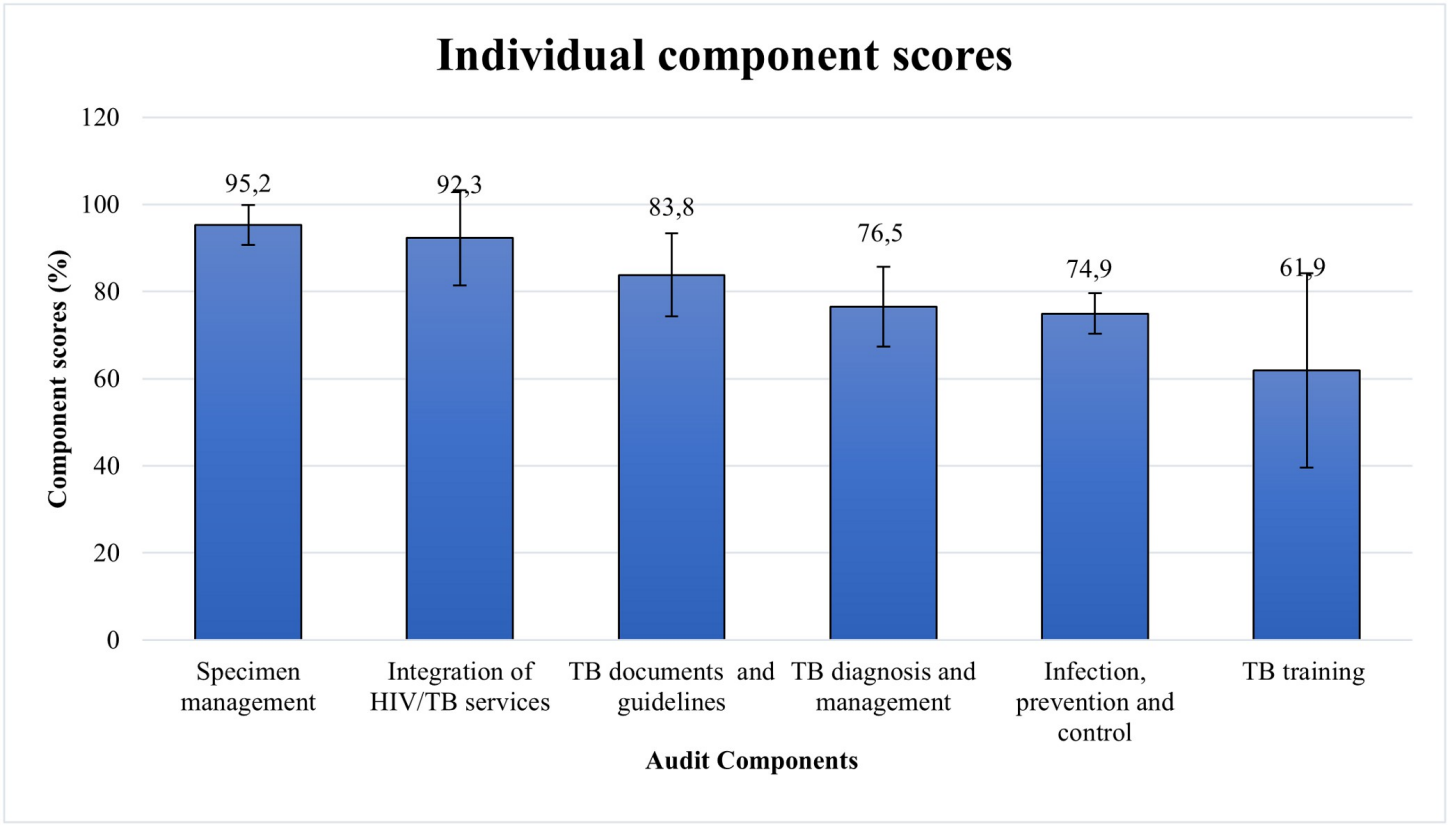
Averages for each audited component of TB diagnostic services in eThekwini, 95% confidence intervals shown with error bars.

The consolidated scores for each component were determined by averaging the scores from the respective components for all clinics in Table 2. The resulting scores for components, specimen management, integration of HIV/TB services, TB policies and guidelines, TB diagnosis and management, Infection, prevention and control, and TB training, are depicted in Fig 2. The audited clinics showed excellent performance for specimen management and integration of HIV/TB services, with 95.2% (95% CI: 90.6–99.8) and 92.3% (95% CI:81.4–103.3), respectively. The remaining audit components performed moderately; TB training had the lowest score of 61.9% (95% CI: 37.5–82.1). The performance of TB training was significantly different from specimen management, TB documents and guidelines, and integration of TB/HIV services, respectively (p < 0.05).

#### Specimen management

This was the best-performing component among all clinics with a total score of 95.2% (95% CI: 90.6–99.8). Only the Southern and Central regions failed to achieve a score of 100% compliance, each with two clinics that scored a 75%. Four clinics could not locate their standard operating procedures (SOPs) for specimen collection. All clinics were compliant in the other areas of specimen management in which sputum was stored under the correct conditions and collected from each facility at least once daily.

#### Integration of TB/HIV services

This service area recorded the second-highest score at 92.3% (95% CI: 81.4–103.3). Every clinic, except two, received a perfect score of 100% for integrating TB/HIV services. All the compliant clinics provided HIV tests to all TB patients and appropriately managed all patients with HIV/TB co-infection according to the national guidelines. One of the clinics that did not fully comply had an HIV/TB coinfected patient who was receiving antiretroviral therapy (ART) from a private doctor. The other non-compliant clinic had a TB patient who was a defaulter and not receiving any HIV care because they still required a referral letter from their previous facility.

#### TB diagnosis and management

This audit component earned a moderate score of 76.5% (95% CI: 67.3–85.7). Individual clinic scores ranged from 40% to 100%. The western region was the only one that excelled, with a combined score of 91.5%. The shortcomings of this component were turnaround time for gene Xpert results and the lack of identification and screening of close contacts of those with active TB. Twelve clinics failed to meet the <48h turnaround time for Gene Xpert results; however, Labtrak results were returned in less than 24h, but these facilities received them after three days. The other nine clinics returned TB test results within two days. Eight facilities failed to identify and screen contacts of patients who had tested TB positive because patients either provided the wrong contact details or failed to give any contact information. All clinics were compliant with using Gene Xpert to diagnose patients, initiating treatment promptly, and investigating the progression of the disease using acid-fast bacillus testing.

#### Infection prevention and control (IPC)

IPC achieved the second-lowest rating with a score of 74.9% (95% CI: 70.3–79.5). Individual clinic scores ranged from 61% to 93%. The most prominent causes for concern were patient waiting areas and the failure by presumptive TB patients and TB nurses to wear surgical masks and N-95 respirators, respectively. Eleven clinics had waiting areas that were small, congested, and lacked adequate ventilation; the waiting areas in the other ten clinics were either outside or had access to fresh air through open windows and doors. Similarly, eleven clinics did not require presumptive TB patients to wear surgical masks. However, ten did supply surgical masks to coughing patients when masks were available. The remaining ten clinics required all patients entering the facility to wear masks. Only one clinic had a TB nurse who wore an N-95 respirator during patient consultations. Nurses at the other twenty clinics either had no access to N-95 respirators due to stockouts, opted for surgical masks instead, or wore nothing due to the heat and discomfort of wearing a mask. On the other hand, all clinics had a designated coughing area away from other patients, with two clinics having a formal coughing booth with a filtration system. Moreover, all but one clinic separated coughing patients and fast-tracked them for TB evaluation.

#### TB documents and guidelines

This audit component earned a moderate score of 83.8% (95% CI: 74.3–93.3). Eight clinics achieved a score of 100%, most of those coming from the western and central regions. The remaining 13 clinics had scores ranging from 14% to 86%. Diagnosis and screening flowcharts and TB posters were notably missing from many clinics. Twelve clinics stated that moving between different consultation rooms was the main reason for the misplaced documentation. During the audit, another clinic had a wing under construction, resulting in many misplaced items, including TB documents. Conversely, all but one clinic had copies of the relevant TB guidelines, and every clinic had either a manual or electronic method of reporting diagnosed TB cases.

#### TB training

TB training was the audit component with the lowest score of 61.9% (95% CI: 37.5–82.1) among all the audited clinics. The western region was the only region that scored 100% in this area, while the central region scored the lowest with only 40%. Eight clinics indicated no staff had received new TB training in the previous 24 months. The two reasons for this were the high staff turnover rate and staff shortages that made it difficult for nurses to leave work to attend offsite training. The remaining 12 clinics had a perfect score (100%) in this area. The TB training received was broad covering topics like the TB cascade of care, up-and-coming diagnostic tools, and loss to follow-up.

### Relationship between clinic characteristics and audit scores

The association between the audit scores and the clinic characteristics (TB headcount and number of TB patients screened) was investigated. The results showed a weak to moderate correlation between component scores and clinic characteristics with no statistical significance (p > 0.05). Assessing the association between region audit scores and clinic characteristics turned up similar results, showing that the numbers of screened patients and clinic headcount were not significantly associated with the audit scores achieved by each region, respectively. Similarly, a linear regression of these variables revealed no relationship between the selected clinic characteristics and the component scores, with R-squared values of less than 1% and no statistical significance.

## Discussion

Facility audits in four different regions of eThekwini were conducted to evaluate the quality TB of diagnostic services at PHCs. The clinic ratings by region were primarily moderate, with only the western region securing an excellent overall score. Moreover, scores obtained by the highest and lowest-performing regions were significantly different from each other. However, no statistically significant relationships were established between audit scores and clinic characteristics. Assessment of individual component scores showed specimen management and the integration of TB/HIV services to have the highest scores. On the contrary, IPC and TB training had the lowest scores. Other notable areas of concern were times for reporting Xpert results and failing to identify and screen close contacts of TB patients.

To our knowledge, no studies from South Africa have looked at the quality of TB services since the COVID-19 pandemic. The audit showed that specimen management was the component that received the highest score. This is good because mishandling and improper storage of sputum can affect its quality, leading to incorrect diagnoses. Some of our study findings were congruent with those from another audit conducted in South Africa, which reported HIV counseling and testing for TB patients as one of the highest audit scores [21]. Integrating HIV/TB services at public healthcare facilities is relatively new and has experienced implementation challenges [32, 33], which led to missed opportunities to care for TB patients appropriately [34]. As such, we did not anticipate this area performing well; however, the targeted quality improvement interventions that have been trialed at PHCs in recent years may be the reason for the high quality of HIV/TB services found in this study [20].

Our study also identified areas of weaker compliance with national standards. Similar research has shown that poor adherence to TB guidelines is often linked to high work volumes [21]. In contrast, our findings showed no associations between the number of patients seen and the audit scores obtained. However, findings from Ethiopia and South Africa were comparable to ours in that high staff turnover formed reasons for the lack of recent training among staff [33, 35]. Ironically, continuous education can be an effective tool for improving the quality of TB services [34]. Adequate training has been linked to better implementation of IPC measures, another barrier to quality identified by this study [36]. Although many facilities adhered to WHO IPC standards by triaging coughing patients and separating them from others [37], we also found that clinic infrastructure resulted in overcrowded waiting areas and inadequate ventilation; moreover, there was a poor supply of masks/respirators, which led to limited mask use. The same challenges burdened health systems during the COVID-19 pandemic [38, 39]. Poorly implemented IPC measures depleted healthcare workers and perpetuated nosocomial infections [40]. Therefore, addressing these IPC challenges serves a dual purpose of improving TB control efforts and strengthening pandemic preparedness.

A cornerstone of the end TB strategy by the WHO is early diagnosis and treatment initiation [18]. Our findings showed potential barriers to both, specifically through Xpert turnaround time and failure to identify and screen close contacts of TB patients. In South Africa, the introduction of Xpert for TB diagnosis has increased the detection of TB by providing results in less than two hours [41], however, its placement at centralized laboratories delays treatment initiation by returning results to the clinics after several days [21, 42]. This was also true for our results. To this end, the National Health Laboratory Services has introduced automated text messages for presumptive TB patients for faster linkage to care [43]. However, this is still in its infancy and has had limited success as of February 2023 [4]. Thus, improving the quality of care needs to look at optimizing the current strategies by working toward delivering same-day diagnosis at the point of care. Indeed, prompt diagnosis reduces the development of more severe forms of TB and curbs community transmission; likewise, rapidly detecting close contacts of TB patients can also reduce the potential for community transmission. However, our findings identified this as another barrier to the quality of TB care. Contact tracing is known to be poorly implemented in resource-limited settings [18, 44] but is a better approach than passive case finding for managing TB [45]. Thus, it requires optimization at PHCs where uptake is mediocre at best.

The study was not without limitations. To assess TB diagnosis and management as well as integration of TB/HIV service a random patient file from each facility was chosen. The number of files was small compared to the number of patients these facilities saw. Therefore, it may not be a true reflection of the two audit components and thus cannot be generalized to the rest of the PHCs in eThekwini. Therefore, a larger study investigating these two parameters would be able to yield more precise estimates. Moreover, while we reported on the high quality of HIV/TB integration services, our research only accounted for patients receiving TB care, which means the HIV program could be missing opportunities to screen and test presumptive TB cases.

### Recommendations

Some of the main barriers to quality of TB care was the need for recent health education on TB among staff, better adherence to IPC measures, turnaround time for results, and contact tracing; based on those we recommend the following: Equipping all staff members working at clinics with TB training so to maintain trained staff. To this end, training can be used to reinforce IPC practices. However, the training should include practical aspects to address the know-do gap. In many cases, the nurses in our study knew the importance of wearing N-95 respirators but did not use them. Furthermore, all patients at PHCs should be mandated to wear masks. This is an effective way of preventing nosocomial infections. COVID-19 has already set a precedent for this and should be leveraged by all health facilities. This would also remove the financial burden from the health system to provide masks for presumptive TB cases, and these funds could be reallocated to purchasing N-95 respirators instead. Moreover, making all patients wear masks will remove any feelings of stigma and discrimination that TB patients may experience. Facilities should use natural ventilation by opening windows and doors. This is an inexpensive way of disrupting the TB transmission cycle by introducing airflow into overcrowded facilities. Using natural airflow over mechanical ventilation is also advantageous, as the country regularly experiences scheduled blackouts that do not always make mechanical ventilation possible. Since COVID-19 ignited the development of novel diagnostic tools, similar efforts should be used to develop a true point-of-care test for TB that would make same-day treatment initiation possible. Authors from Malawi have explored patient-delivered screening interventions for improving contact tracing and initiation of preventative therapy [46]. Similar interventions should be examined for the South African context to assist with the low uptake of contact screening at clinics. Lastly, continuous quality assessments at PHCs should continue to determine areas that need to be addressed and establish the efficiency of any newly implemented practices.

## Conclusions

We found that the overall quality of TB diagnostic services was moderate throughout PHCs in the eThekwini district. While specimen management and integration of HIV/TB services excelled, some IPC measures, TB training, reporting of results, and contact tracing, performed poorly. These underperforming areas have the potential to undermine TB management strategies that are currently in place and, therefore, will need to be adequately addressed to continue making progress in the fight against TB, especially in light of the COVID-19 pandemic that has slowed down many of the gains achieved in recent years.

## Supporting information

S1 Table Quality of TB diagnostic services at primary healthcare clinics in eThekwini district, South Africa: Data collection tool. (DOCX)

## Abbreviations

ANOVA: Analysis of Variance
ART: Antiretroviral therapy
IPC: Infection, prevention, and control
NDoH: National Department of Health
NTP: National Tuberculosis Program
PHCs: Primary Healthcare Clinics
PI: Primary Investigator
SDGs: Sustainable Development Goals
TB: Tuberculosis
USAID: United States Agency for International Development
WHO: World Health Organization
