# Supplementary material for: Quality of TB diagnostic services at primary healthcare clinics in eThekwini district, South Africa
Source: PLoS One. 2024 Jul 18;19(7):e0307149. doi: 10.1371/journal.pone.0307149 (PMC11257344; doi:10.1371/journal.pone.0307149)
Supplement: S1 Table — (DOCX) [file pone.0307149.s001.docx]

**Quality of TB diagnostic services at primary healthcare clinics in eThekwini district, South Africa: Data collection tool**

| ***Facility Identification*** | | | | |
| --- | --- | --- | --- | --- |
| Facility Name |  | | | |
| Location of facility |  | | | |
| ***Facility Characteristics*** | | | | |
| TB service availability | \| Outpatient only  Inpatient only  Both inpatient and outpatient \| \| --- \| | | | |
| ***Facility Visit*** | | | | |
| Visit Date | Interviewer ID and Name | Result | | |
| ___/___/______ | Name: | Completed  Partially completed  Respondent unavailable  Facility refused  Postponed  Other (specify)  __________________________ | | |
| ***Availability of TB diagnostic services*** | | | | |
|  |  | **Yes** | **No** | **Comment** |
|  | Does this facility provide TB screening and diagnosis services? |  |  |  |
|  | Typically, how many days per week are TB-related services offered? |  |  |  |
|  | How many service points *(i.e. number of places within the facility)* deliver TB-related services in the facility? |  |  |  |
|  | Now, I will ask if the facility provides certain TB-related services. For each service, I want to know whether this facility ever offered the service. | | | |
|  | Diagnosis of tuberculosis based on any type of specimen testing (smear, culture, rapid test) |  |  |  |
|  | Diagnosis of tuberculosis based on clinical symptoms |  |  |  |
|  | TB medicines given directly to patient by health provider |  |  |  |
| ***Integration of TB/HIV services*** | | | | |
|  | HIV testing and counseling for TB patients |  |  |  |
|  | ART for TB/HIV co-infected patients |  |  |  |
|  | CPT for TB/HIV co-infected patients |  |  |  |
|  | Viral load testing for TB/HIV co-infected patients |  |  |  |
|  | TH/HIV+ patient enrolled for HIV care  (SCR opened and CD4 recorded?) |  |  |  |
|  | Do you use the following methods for diagnosing TB in this facility? | **Yes** | **No** | **Comment** |
|  | GeneXpert MTB/RIF for patient |  |  |  |
|  | Are smears for AFB taken at 7 weeks/ Documented |  |  |  |
|  | Access to line-probe assays (LPAs) for non-converters |  |  |  |
|  | are smears for AFB taken at 11 weeks |  |  |  |
|  | Are second and third smears taken |  |  |  |
|  | If yes recorded in register |  |  |  |
|  | ***TB diagnosis and Management* [TB Focal Person or Lab Personnel]** | | | |
|  |  | **Yes** | **No** | **Comment** |
|  | Does the facility send specimens outside of the facility for TB testing? |  |  |  |
|  | Does the facility keep records of results of sputum tests? |  |  |  |
|  | Does the facility keep records of the results from the quality control (internal or external) procedures? |  |  |  |
|  | What type of TB test services are performed at the facility? |  |  |  |
|  | Ziehl-Neelsen testing for TB (AFB) |  |  |  |
|  | Ziehl-Neelsen testing for TB (AFB) Turnaround time ≤48h |  |  |  |
|  | Xpert® MTB/RIF diagnostic testing for TB |  |  |  |
|  | Xpert® MTB/RIF diagnostic testing for TB Turnaround time ≤48h |  |  |  |
| \|  \| ***Policies, Protocols, and Guidelines* [In-charge or TB Focal Person]** \| \| --- \| --- \| | | | | |
|  | Next, I’d like to assess the availability of copies of approved and required protocols, policies, and messages on TB information available at the facility: | **Yes** | **No** | **Comment** |
|  | The national TB management and control guidelines |  |  |  |
|  | Guidelines and procedures for quality control (either internal or external) for the specimens assessed in this facility |  |  |  |
|  | The National Guidelines for clinical management of TB/ HIV related conditions |  |  |  |
|  | Protocol or guideline of essential drug list or essential medicines list |  |  |  |
|  | Guidelines related to MDR-TB diagnosis and treatment (or identification of need for referral) |  |  |  |
|  | Flowcharts or algorithms on TB screening |  |  |  |
|  | Flowcharts or algorithms on TB diagnosis |  |  |  |
|  | TB posters on walls, leaflets, brochures, and pamphlets in local languages for distribution, i.e. educational materials about TB available |  |  |  |
|  | ***TB Training among staff*[In-charge or TB Focal Person]** | | | |
|  | Did any providers of TB services at this facility receive new or refresher training in the following topics in the last 24 months? If yes what did the training cover? | **Yes** | **No** | **Comment** |
|  | ***Drug Regimens* [TB Focal Person]** | | | |
|  | Do staff or personnel initiate and prescribe drug regimens in line with existing national protocol for: | **Yes** | **No** | **Comment** |
|  | Newly diagnosed patients |  |  |  |
|  | Re-treatment patients |  |  |  |
|  | Adult contacts - Preventive treatment for TB infection (INH + Pyridoxine)/ Screened and started on treatment |  |  |  |
|  | ***Specimen Management* [Lab Personnel or TB Focal Person]** | | | |
|  | How is sputum collected? | **Yes** | **No** | **comment** |
|  | Are (Standard Operating Procedures) SOPs for specimen collection available? |  |  |  |
|  | Does the facility have the contact details of their laboratory? |  |  |  |
|  | Are the approved laboratory request forms available? |  |  |  |
|  | Is there an up-to-date specimen dispatch list? |  |  |  |
|  | Were there any stock-outs of specimen supplies in the past 6 months? |  |  |  |
|  | Does specimen transportation to the laboratory occur (within 48 hours) |  |  |  |
|  | Does the facility use a cooler box reserved for transportation of specimens? |  |  |  |
|  | ***Adequacy of Infection Prevention Measures* [Infection Control Focal Person or TB Focal Person]** | | | |
|  | I’m going to ask about infection prevention measures and then I’d like to see the supplies used for infection control | **Yes** | **No** | **comment** |
|  | A staff member has been designated as an infection prevention and control focal point with specifically articulated duties |  |  |  |
|  | TB infection prevention and control practices are followed according to national guidelines |  |  |  |
|  | Patients are routinely asked about cough when entering the facility |  |  |  |
|  | PLEASE **OBSERVE** TO SEE IF THE FOLLOWING RESOURCES/SUPPLIES USED FOR INFECTION CONTROL ARE AVAILABLE IN THE FACILITY WHERE TB PATIENTS ARE RECEIVING SERVICES ON THE DAY OF ASSESSMENT. **[ASK TO SEE THE ITEMS]** |  |  |  |
|  | An updated and approved infection prevention and control plan is available for the facility |  |  |  |
|  | A TB infection prevention and control risk assessment is completed at least annually |  |  |  |
|  | There is a facility reporting system for all patients diagnosed with TB and referred for treatment (in accordance with national policies) |  |  |  |
|  | Cough triage is implemented (patients that are coughing are separated from others and fast-tracked for evaluation) |  |  |  |
|  | A cough monitor or other designated person assists with separation and triage of coughing patients |  |  |  |
|  | Supplies are available to coughing patients (tissues, masks, etc.) |  |  |  |
|  | Specimens are collected in any of the following designated areas: |  |  |  |
|  | Outside the service delivery point |  |  |  |
|  | Away from other patients |  |  |  |
|  | In a well-ventilated area |  |  |  |
|  | Patient waiting areas are outside or have access to fresh air continuously |  |  |  |
|  | Surgical masks are available and worn by presumptive and TB patients |  |  |  |
|  | N-95 and FFP2 respirators are readily available for staff |  |  |  |
|  | Staff have been trained on proper fit of respirators |  |  |  |
|  | N95 worn by staff |  |  |  |
|  | Patients wearing masks at facility |  |  |  |
